# Prognostic relevance of arrhythmic and QTc burden in takotsubo cardiomyopathy: A systematic review and meta‐analysis

**DOI:** 10.1002/joa3.70138

**Published:** 2025-07-16

**Authors:** Ankit Hanmandlu, Jyothik Varun Inampudi, Mohammad Hamza, Prakash Upreti, Abdul Rasheed Bahar, Jawad Basit, Sivaram Neppala, Himaja Dutt Chigurupati, Rohit Goru, M. Chadi Alraies

**Affiliations:** ^1^ Wayne State University/Detroit Medical Center Department of Internal Medicine Detroit Michigan USA; ^2^ Guthrie Medical Group Cortland New York USA; ^3^ Sands‐Constellation Heart Institute Rochester Regional Health Rochester New York USA; ^4^ Department of Medicine Rawalpindi Medical University Rawalpindi Pakistan; ^5^ UT Health Science Center at San Antonio San Antonio Texas USA; ^6^ New York Medical College at Saint Michael's Medical Center Newark New Jersey USA; ^7^ Wayne State University School of Medicine Detroit Michigan USA; ^8^ Wayne State University/Detroit Medical Center Cardiovascular Institute Detroit Michigan USA

**Keywords:** atrial arrhythmias, life‐threatening arrhythmias, QTc, takotsubo cardiomyopathy

## Abstract

**Introduction:**

Takotsubo cardiomyopathy is characterized by stress‐induced systolic dysfunction of the left ventricle that is largely reversible and not related to coronary ischemia. Patients with Takotsubo cardiomyopathy can often develop concurrent arrhythmias and prolonged QTc interval, which have been shown to contribute to significant morbidity and mortality in prior retrospective studies. Hence, we conducted a comprehensive systematic review and meta‐analysis to characterize the risk of atrial arrhythmias, life‐threatening arrhythmias, and QTc prolongation on clinical outcomes in patients with Takotsubo cardiomyopathy.

**Methods:**

We searched PubMed and EMBASE databases from inception to April 2024. A total of 13 studies were eligible for data collection across the three subgroups.

**Results:**

Atrial and life‐threatening arrhythmias in Takotsubo cardiomyopathy have an increased risk of in‐hospital mortality and cardiogenic shock, a finding that was not observed in patients with prolonged QTc. Upon univariate analysis of the QTc subgroup, younger age and the presence of ST‐Elevation were identified as additional negative prognostic indicators of adverse outcomes in Takotsubo cardiomyopathy.

**Conclusion:**

Our study demonstrates the increased risk of different arrhythmic profiles in Takotsubo cardiomyopathy. Further investigation is needed to identify pathophysiological mechanisms and tailored anti‐arrhythmics to improve outcomes in this unique subset of Takotsubo patients.

## Graphical abstract

The objective of the study was to evaluate the prognostic implications of the arrhythmic and QTc burden in Takotsubo cardiomyopathy. We found that atrial arrhythmias and ventricular arrhythmias significantly increased in‐hospital morbidity and mortality, while prolonged QTc only increased in‐hospital morbidity. It is critically important to recognize this high‐risk and unique subset of Takotsubo patients in order to optimally treat and improve clinical outcomes.

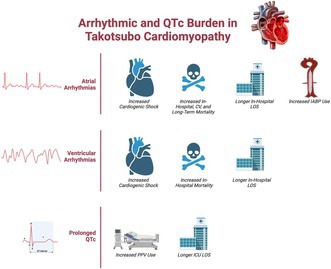

## 1 INTRODUCTION

Takotsubo cardiomyopathy (TCM) is an acute onset, temporary, non‐ischemic cardiomyopathy characterized by systolic dysfunction of the ventricle.^1^ TCM is usually triggered by emotional and physical stress; however, it can also occur in the absence of any stress factor.^2^ Possible pathophysiological mechanisms involved in the development of TCM include coronary vasospasm and myocardial stunning due to catecholamines.^3^ Although the true incidence of TCM is unknown, it is postulated to be 15–30 cases per 100,000 per year, with an estimated in‐hospital mortality of up to 5%.^4^ The apical form is the most prevalent variant (81.7%), followed by the mid‐ventricular, basal, and focal subtypes.^5^ Despite its high clinical significance, there are no evidence‐based treatment strategies for TCM due to the lack of randomized controlled trials, and its management has been mainly supportive.^6^

Arrhythmias commonly co‐occur in TCM patients. These arrhythmias can include life‐threatening arrhythmias (LTAs) such as ventricular tachycardia (VT), ventricular fibrillation (VF), complete atrioventricular (AV) block, pulseless electrical activity, asystole, as well as more benign rhythms like atrial fibrillation and flutter (AAs).^7^, ^8^, ^9^ Furthermore, TCM can also lead to temporary abnormalities in ventricular repolarization, leading to prolonged QTc and the establishment of a proarrhythmic state.^10^

This study specifically focuses on AAs, LTAs, and QTc prolongation due to their associations with increased morbidity and mortality, but uncertain clinical significance.^11^, ^12^, ^13^ A better understanding of the arrhythmic and QTc burden in TCM can help with early risk assessment, increased clinical surveillance, and prompt intervention to improve actionable endpoints. Hence, we conducted a large systematic review and meta‐analysis to evaluate the clinical outcomes of patients with LTAs, AAs, and prolonged QTc in TCM patients.

## 2 METHODS

The details of the methods for this systematic review and meta‐analysis used the recommendations in the Preferred Reporting Items for Systematic Reviews and Meta‐Analysis (PRISMA),^14^ Assessing the Methodological Quality of Systematic Reviews‐2 (AMSTAR‐2),^15^ and Meta‐analysis of Observational Studies in Epidemiology statements and guidelines.^16^ Relevant checklists can be found in Supplemental S1–S2.

### 2.1 Search strategy

Two investigators (A.H. and J.I.) searched both PubMed and EMBASE databases for publications from inception to April 2024 using medical subject heading terms (MeSH) terms and keywords combined with Boolean operators “OR” and “AND” for “arrhythmia”, “QTc”, “torsade de pointes”, “Takotsubo”/“Apical Ballooning Syndrome”, “incidence”, “mortality”/“death”, “prognosis”/“survival” and “outcome.”

Moreover, upon reviewing the articles identified through the search strategy, we hand‐searched the reference lists of systematic reviews/meta‐analyses and review articles to locate any missed studies. Our search included full‐text articles in English or with readily available English translations containing studies on human adults. The full search strategy is detailed in Supplemental S3.

### 2.2 Inclusion and exclusion criteria

A prespecified criterion was developed, and each study was evaluated for inclusion based upon (1) observational and experimental studies, (2) with arrhythmic burden in Takotsubo, and (3) reporting predictive, prognostic, and outcome‐related variables.

The diagnosis of Takotsubo Cardiomyopathy was made based on adherence to the Mayo Clinic criteria, Madias criteria, or European Society of Cardiology expert consensus/task force statements. All references using the National Inpatient Sample (NIS) or reporting Takotsubo Cardiomyopathy solely using the International Classification of Diseases (ICD) codes were excluded. We did this to avoid the misclassification of Takotsubo diagnosis related to large data sources, and to ensure diagnostic validity and consistency across included studies.

### 2.3 Study selection

**FIGURE 1:**
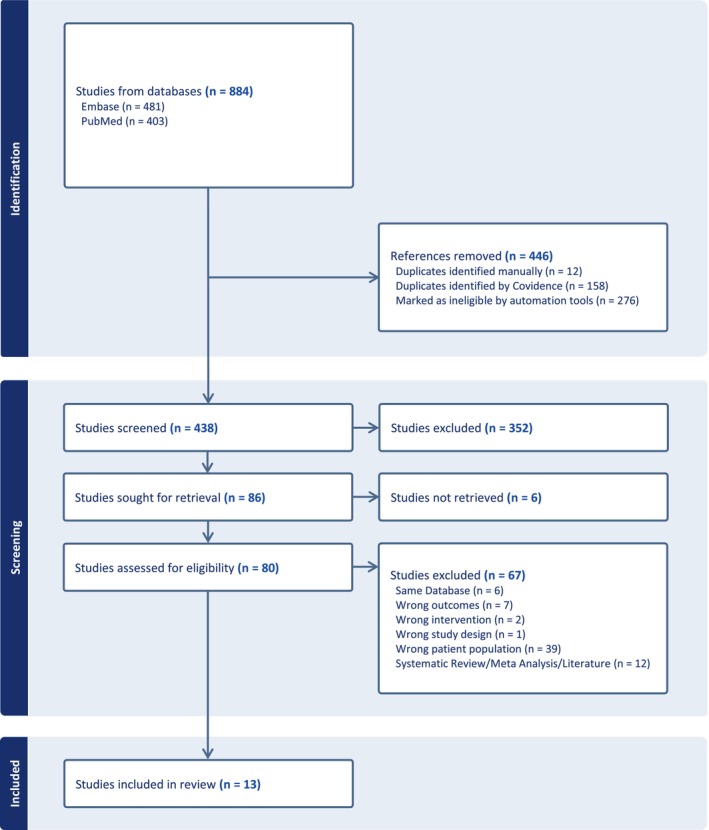
PRISMA diagram showing the study selection process.

There were 884 studies that returned upon application of the search strategy, 481 from EMBASE and 403 from PubMed (Figure 1). Of these, 446 references were removed. This included 276 that were auto‐marked ineligible by COVIDENCE and 170 duplicates. Importantly, any study eliminated by COVIDENCE automation tools was also manually screened to prevent omission of relevant studies.

Altogether, 438 articles underwent title and abstract screening, and 352 were excluded. Of these, 86 studies were identified and subsequently selected for full review. All studies were reviewed to ensure no overlapping patient databases were included in the same subgroup analysis. The senior author (M.C.A.) reviewed and oversaw the screening process.

### 2.4 Data extraction

Two investigators (A.H. and J.I.) independently reviewed the selected studies and evaluated them for inclusion according to the predefined criteria. Any disagreements were resolved with a consensus opinion. In total, we selected 13 studies for data extraction. Of those excluded, 39 articles assessed a different patient population, 12 articles were systematic reviews/meta‐analyses or literature reviews, 7 articles assessed different outcomes, 6 articles did not have readily available full English texts (not retrieved), 2 evaluated different interventions, 6 articles evaluated the same overlapping Takotsubo registry, and 1 study performed a different study design. We extracted data from 13 studies under three separate subgroups: atrial arrhythmias, life‐threatening arrhythmias, and QTc burden. The components extracted include the following: study design, baseline patient characteristics, diagnostics on presentation, treatment, and outcomes. This was organized in Microsoft Excel. Primary outcomes included in‐hospital mortality, while secondary outcomes evaluated cardiogenic shock, thromboembolism, intraaortic balloon pump use (IABP), LTAs, length of stay (LOS), long‐term mortality, positive pressure ventilation (PPV) use, and inotrope use.

### 2.5 Data analysis

We performed statistical analysis with CRAN‐R software (The R Foundation for Statistical Computing, Vienna, Austria). Means with standard deviations and frequency of baseline demographics and comorbidities were reported. Continuous variables were assessed using a *meta‐cont* module and an inverse variance method to calculate the pooled standardized mean difference (SMD). Moreover, categorical variables were evaluated by a *meta‐bin* module using the inverse variance random effects model to calculate the pooled odds ratio (OR). A *p*‐value <0.05 was considered statistically significant, and results were reported with 95% confidence intervals (95% CI). Analysis of study heterogeneity was performed using Higgins *I*‐squared (*I* ^2^) statistic. Mild heterogeneity was defined as <50%, moderate heterogeneity was defined as between 50% and 75%, and severe heterogeneity was defined as >75%.

### 2.6 Risk of bias

We performed a risk of bias assessment using the Newcastle‐Ottawa Scale.^17^ Each study included in our meta‐analysis received a score of ≥7/9, which demonstrated that all studies were of high quality with a low risk of bias (Supplemental S5). Although ClinicalTrials.gov mostly contains interventional trials, some prospective observational studies are also listed, and therefore we searched it to further minimize the possibility of publication bias. The search turned up no relevant unpublished or selectively reported studies.

## 3 RESULTS

### 3.1 Summary of studies

The study search encompassed 13 articles conducted within three sub‐analyses published between 2011 and 2023.^9^, ^11^, ^12^, ^13^, ^18^, ^19^, ^20^, ^21^, ^22^, ^23^, ^24^, ^25^, ^26^ Altogether, the QTc sub‐analysis included 474 patients, the AA sub‐analysis included 2185 patients, and the LTA sub‐analysis contained 1396 patients. Overall, this accounted for 4055 patients. However, two studies^9^, ^20^ with 214 patients used the same database but were not included in the same subgroup analysis. Most of the studies were retrospective, with few being prospective and/or having prospective components. Study characteristics, important comorbidities, and relevant diagnostic information are shown in Tables 1, 2, 3.

**TABLE 1:** Baseline characteristics of included studies with and without atrial arrhythmias.

Variables (AA/No AA)	Stiermaier 2017	Jesel 2019	El‐Battrawy 2021
Study type	Retrospective	Retrospective	Retrospective
Total patients	97/290	53/161	112/1472
Age (SD)			
AA	78 ± 8.38	70 ± 12	73.8 ± 12.2
No AA	71.25 ± 4.33	68 ± 12	66.1 ± 13
Female	66/262	39/135	95/1334
HTN	82/227	28/93	93/918
DM	28/61	12/35	28/187
Smoking	16/60	13/31	13/278
TCM subtypes			
Apical	84/214	33/110	100/1198
Midventricular	13/71	20/40	—
Basal	0/4	0/1	—
STE	—	—	50/640
TWI	—	—	43/605
LVEF %			
AA	37 ± 4.06	35 ± 11	37.2 ± 11.1
No AA	42 ± 4.05	39 ± 11	41.3 ± 11.8

*Note*: AA/without atrial arrhythmias.

Abbreviations: AA, atrial arrhythmia; DM, diabetes mellitus; HTN, hypertension; LVEF, left ventricular ejection fraction; STE, ST‐elevation; TCM, Takotsubo cardiomyopathy; TWI, T‐wave inversions.

**TABLE 2:** Baseline characteristics of included studies with and without life‐threatening arrhythmias.

Variables (LTA/No LTA)	Jesel 2018	El‐Battrawy 2020	Auzel 2016	Pelargonio 2021	Madias 2011
Study type	Retrospective	Prospective	Retrospective	Retrospective	Retrospective/prospective
Total patients	23/191	67/839	9/81	23/70	8/85
Age (SD)					
LTA	66.4 ± 13	71 ± 11	62 ± 17	72 ± 13	64.75 ± 6.62
No LTA	69.4 ± 12.6	70 ± 11	73 ± 12	72 ± 12	67.25 ± 6.64
Female	16/158	56/754	9/78	22/63	5/75
HTN	13/108	46/587	3/38	15/49	5/45
DM	6/41	16/171	1/7	9/19	1/17
HLD	6/76	—	3/23	10/38	1/30
Smoking	—	14/191	1/24	8/22	4/45
TCM subtypes					
Apical	16/137	54/700	3/60	17/55	8/75
Mid ventricular	6/54	13/121	—	4/15	—
Basal	0/1	0/17	—	—	—
Focal	—	0/1	—	1/1	—
Apical sparing	—	—	6/20	—	—
Inverted	—	—	0/1	1/0	—
QTc					
LTA	470 ± 51	459 ± 65	439 ± 46	458 ± 55	602 ± 32
No LTA	461 ± 44	454 ± 89	447 ± 52	448 ± 52	490 ± 15
LVEF %					
LTA	31 ± 10	37.1 ± 9.34	40.4 ± 5.7	43 ± 10	35.75 ± 3.76
No LTA	41 ± 11	40.07 ± 8.81	42.5 ± 9.9	48 ± 10	26.25 ± 4.35

*Note*: LTAs/Without LTAs.

Abbreviations: DM, diabetes mellitus; HLD, hyperlipidemia; HTN, hypertension; LTA, life‐threatening arrhythmia; LVEF, left ventricular ejection fraction; TCM, Takotsubo cardiomyopathy.

**TABLE 3:** Baseline characteristics of included studies with and without QTc prolongation.

Variables (QTc/No QTc prolongation)	Song 2014	Hohneck 2019	Imran 2016	Pinho 2023	Del Buono 2022
Study type	Retrospective	Retrospective	Retrospective	Retrospective	Retrospective
Total patients	50/55	73/32	25/21	43/70	28/77
Age (SD)					
QTc prolongation	63.25 ± 5.48	66.55 ± 11.54	61.4 ± 15.2	66.7 ± 12.9	68 ± 10.16
No QTc prolongation	64.25 ± 5.49	66.69 ± 10.43	57.8 ± 17.2	68.1 ± 10.9	64 ± 10.58
Male	17/12	12/7	9/6	3/3	4/14
HTN	16/14	44/15	19/10	—	18/38
DM	8/10	18/5	5/3	—	7/15
Smoking	3/4	25/9	8/11	—	—
STE	41/35	26/6	5/3	13/24	—
TWI	48/32	73/28	13/10	39/51	—
LVEF %					
QTc prolongation	39.2 ± 7.6	38.3 ± 9.2	29 ± 9.6	—	28.3 ± 7.81
No QTc prolongation	43.5 ± 5.9	38 ± 10.5	25 ± 8.8	—	28.3 ± 7.55

*Note*: Prolonged QTc/Normal QTc.

Abbreviations: DM, diabetes mellitus; HTN, hypertension; LVEF, left ventricular ejection Fraction; STE, ST‐elevation; TWI, T‐wave inversions.

To deal with heterogeneity, we performed sensitivity analysis on clinical outcomes with *I* ^2^ > 50% heterogeneity and at least three studies. Studies such as Song,^21^ Stiermaier,^23^ and Hohneck et al.^13^ had higher event rates, longer hospital stays, prolonged follow‐up, or imbalanced group sizes that disproportionately influenced pooled estimates. These outliers reflect variations in study design and population characteristics that may affect clinical interpretation.

### 3.2 Atrial arrhythmias

The average age was 74.17 and 67.19 in the AAs and without AAs groups, respectively. Most patients were female, 76% in the AA arm and 90% in the without AAs arm. The most common comorbidity was hypertension, which was present in 77.4% of patients with AAs and 64.1% in patients without AAs. The TCM subtypes in the AAs group included 88% apical and 12% midventricular. Contrarily, the without AAs group consisted of 81% apical, 6% midventricular, and <1% basal involvement of TCM. Average LVEF was higher in patients without AAs (41.20%) than in patients with AAs (36.95%).

**FIGURE 2:**
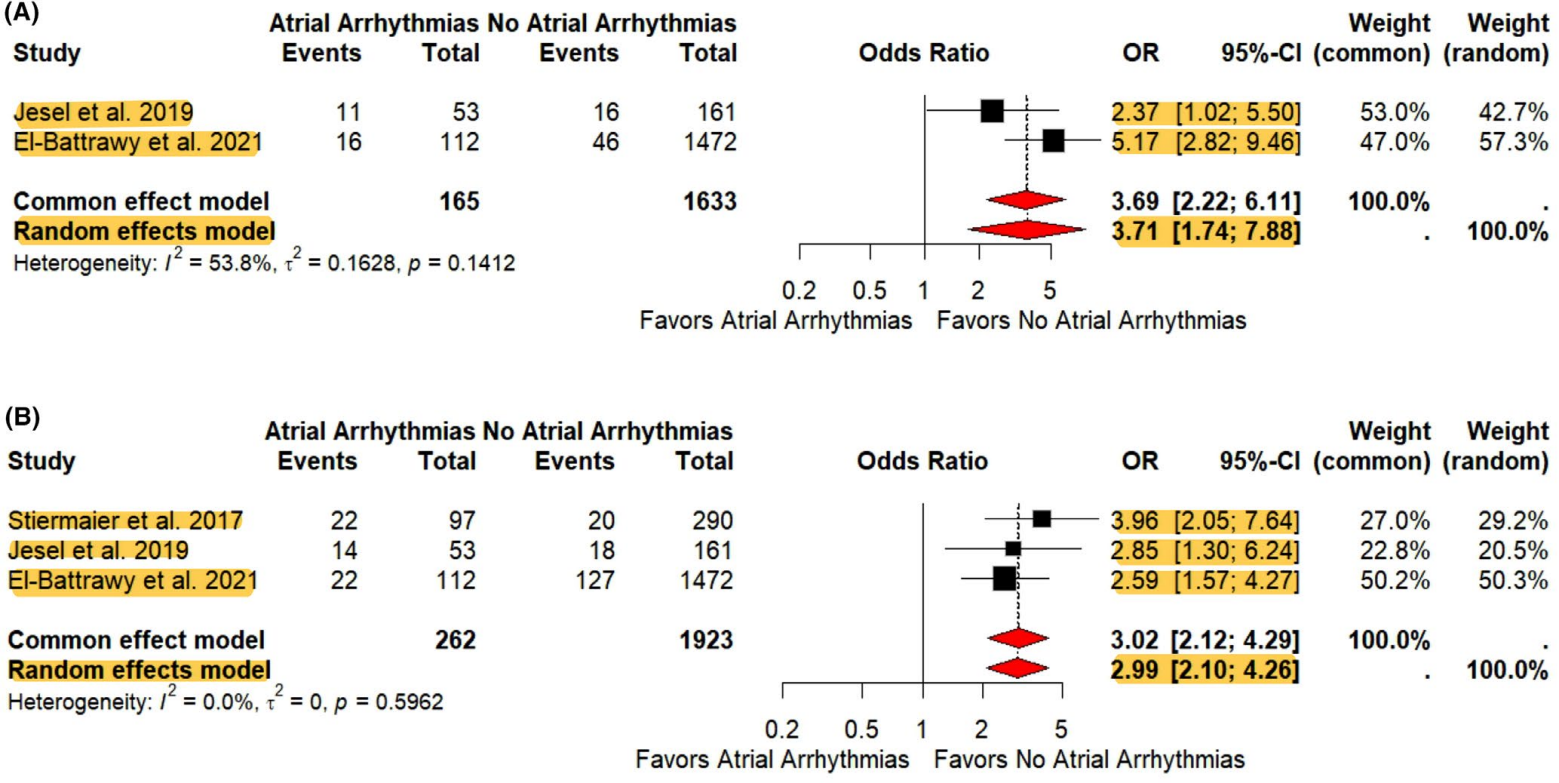
Forest plot showing contribution of atrial arrhythmias on in‐hospital mortality and cardiogenic shock. (A) In‐hospital mortality and, (B) cardiogenic shock, CI = confidence interval, OR = odds ratio. Highlights indicate significant trends within each Forest Plot. Area of the black box correlates to the weight of the study in the meta‐analysis. Red diamonds indicate pooled confidence intervals.

The rate of in‐hospital mortality (OR 3.71, 95% CI 1.74–7.88, *p* = 0.007), cardiovascular mortality (OR 4.39, 95% CI 2.25–8.56, *p* < 0.001), and long‐term mortality (OR 3.30, 95% CI 2.33–4.69, *p* < 0.0001) in patients with AAs was significantly higher than in patients without AAs (Figure 2A and Supplemental S4). The heterogeneity was moderate (*I* ^2^ = 54%), mild (*I* ^2^ = 0%), and mild (*I* ^2^ = 0%) in two studies, respectively. There was a significantly higher risk of cardiogenic shock in the AAs arm compared to the arm without AAs (OR 2.99, 95% CI 2.10–4.26, *p* < 0.0001) with mild heterogeneity (*I* ^2^ = 0%) observed between studies (Figure 2B). Furthermore, the incidence of intra‐aortic balloon pump use in the AAs group was higher than in the non‐AAs group (OR 2.58, 95% CI 1.39–4.79, *p* < 0.0026), with mild heterogeneity between studies (*I* ^2^ = 0%), illustrated in Supplemental S4. Lastly, length of stay (LOS) was longer in patients with AAs than in those without AAs (SMD 0.91, 95% CI 0.46–1.36, *p* < 0.0001) as shown in Supplemental S4. There was severe heterogeneity between 3 studies (*I* ^2^ = 89%).

Sensitivity analysis was performed to address the severe heterogeneity found when evaluating length of stay. When taking out Stiermaier et al.,^23^ the *I* ^2^ went to 0% (mild heterogeneity). This was likely secondary to all TCM patients requiring admission into the coronary care unit for at least 24 hours before the decision was made to de‐escalate care, inadvertently influencing length of stay in both the AAs and without AAs subgroups.

### 3.3 Life‐threatening arrhythmias

The average age was 69.36 and 70.03 in the LTAs group and without the LTAs group, respectively. The female gender comprised 83% in the LTA arm and 89% in the without LTAs arm. Hypertension was the most common comorbidity present in 63% of patients with LTAs and 65% of patients without LTAs. Apical TCM was the most frequent subtype and was lower in the LTAs group (75%) than in those without the LTAs group (81%). Less frequent subtypes included mid‐ventricular (18% in LTAs vs. 15% without LTAs) and <1% of basal, focal, inverted, and apical sparing involvement in both groups. The average baseline QTc was 459.43 and 454.21 in the LTAs arm and without the LTAs arm, respectively (SMD 95% CI −0.101 to 0.276, *p* = 0.3641) as shown in Table 4. Average LVEF was largely similar in patients with LTAs (37.21) to those without LTAs (39.88).

**TABLE 4:** Baseline QTc between LTAs and non‐LTAs groups.

Group	Baseline QTc (mean [95% CI])
Life‐threatening arrhythmias (LTA)	459.43 [449.16–469.69]
No life‐threatening arrhythmias (Non‐LTA)	454.21 [447.85–460.56]
Difference (LTA vs. Non‐LTA)	0.0874 [−0.101–0.276]
*p*‐value	0.3641

**FIGURE 3:**
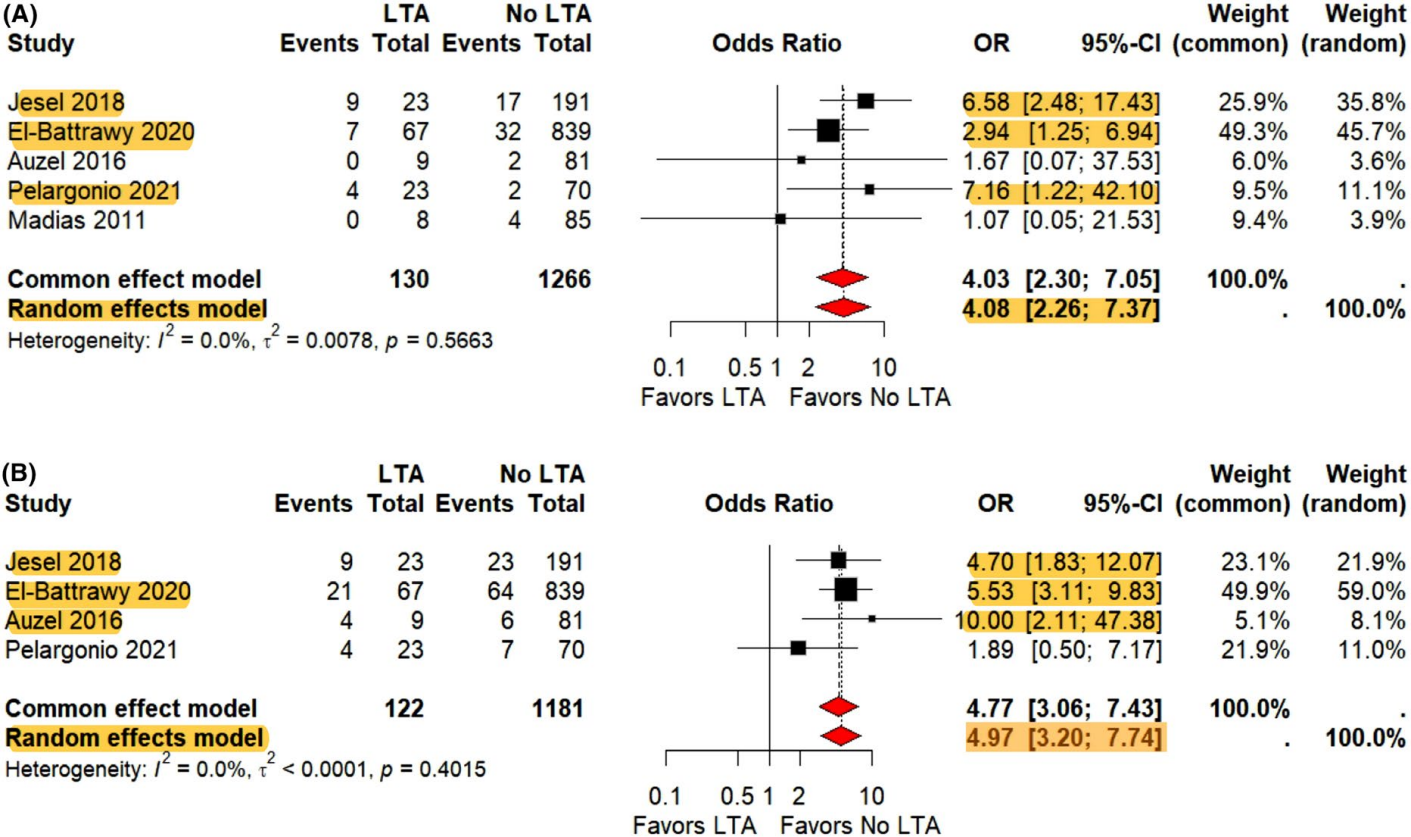
Forest plot showing contribution of life‐threatening arrhythmias on in‐hospital mortality and cardiogenic shock. (A) In‐hospital mortality and, (B) cardiogenic shock, CI = confidence interval, OR = odds ratio. Highlights indicate significant trends within each Forest Plot. Area of the black box correlates to the weight of the study in the meta‐analysis. Red diamonds indicate pooled confidence intervals.

There was a significantly higher rate of in‐hospital mortality (OR 4.08, 95% CI 2.26–7.37, *p* < 0.0001) and cardiogenic shock (OR 4.97, 95% CI 3.20–7.74, *p* = < 0.0001) in the LTA arm compared to the non‐LTA arm (Figure 3A,B). The heterogeneity was mild (*I* ^2^ = 0) for both outcomes between four and five studies, respectively. LOS was reported to be longer in patients with LTAs versus without LTAs (SMD 0.45 95% CI 0.14 to 0.75, *p* = 0.0046) with mild heterogeneity (*I* ^2^ = 39%) between three studies (Supplemental S4). The incidence of thromboembolism was not significantly different in patients with LTAs compared to those without LTAs (OR 2.29, 95% CI 0.84–6.23, *p* = 0.1063) with mild heterogeneity (*I* ^2^ = 0%) between the two studies (Supplemental S4).

### 3.4 QTc changes

The threshold for prolonged QTc differed across all the studies but was at least >430 milliseconds (ms) in men and 450 ms in women (Supplemental S7). The average age was 65.52 and 65.01 in the prolonged QTc and normal QTc arms, respectively. Males were the least predominant gender, comprising 19% of the prolonged QTc group and 16% of the normal QTc group. The most common comorbidity was hypertension, which was prevalent in 44% of patients with prolonged QTc compared to 30% of patients with normal QTc. When evaluating the electrocardiogram, the prolonged QTc group had ST‐Elevation (ST‐E) in 45% and T‐Wave Inversion (TWI) in 90% of patients. In comparison, the normal QTc group had ST‐E in 38% and TWI in 68% of patients, respectively. The average LVEF was 35.64 and 34.13 in the prolonged QTc and normal QTc arms, respectively.

**FIGURE 4:**
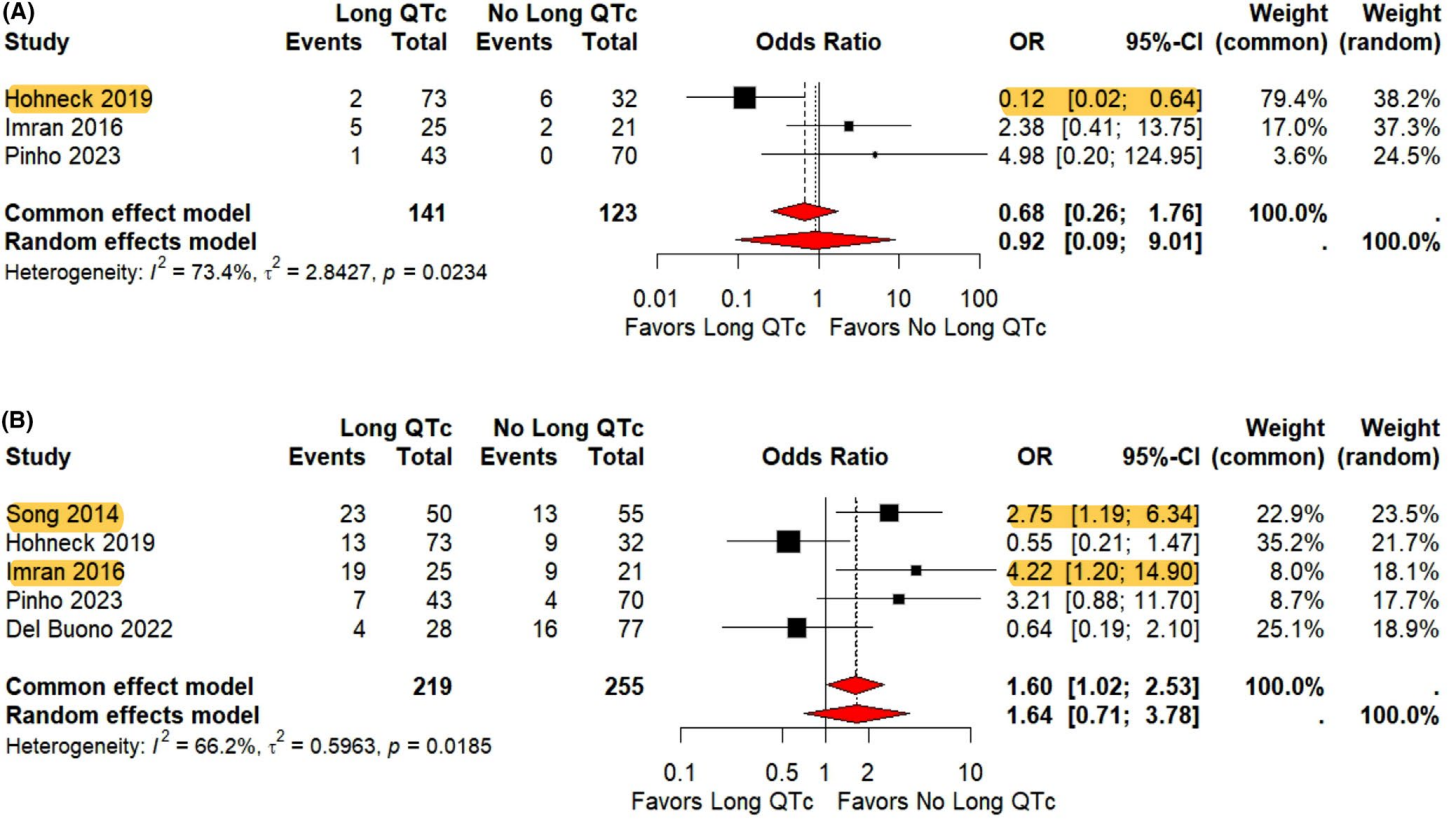
Forest plot showing the contribution of prolonged QTc to in‐hospital mortality and cardiogenic shock. (A) In‐hospital mortality and, (B) cardiogenic shock, CI = confidence interval, OR = odds ratio. Highlights indicate significant trends within each Forest Plot. The area of the black box correlates to the weight of the study in the meta‐analysis. Red diamonds indicate pooled confidence intervals.

There was no statistically significant difference between patients with prolonged QTc and normal QTc in all‐cause mortality (OR 0.78, 95% CI 0.23–2.66, *p* = 0.6945) or in‐hospital mortality (OR 0.92, 95% CI 0.09–9.01, *p* = 0.9406), shown in Figure 4A and Supplemental S4. The heterogeneity was moderate (*I* ^2^ = 64% and 73%) between the three studies, respectively. The rate of cardiogenic shock was not significantly different in the prolonged QTc arm compared to the normal QTc arm (OR 1.64, 95% CI 0.71–3.78, *p* = 0.2500) with moderate heterogeneity (*I* ^2^ = 66.2%) between 5 studies (Figure 4B). The incidence of LTAs (OR 2.17, 95% CI 0.99–4.77, *p* = 0.0540) and inotrope use (OR 1.14, 95% CI 0.45–2.90, *p* = 0.7833) was reported to be statistically insignificant in the prolonged QTc group versus the normal QTc group (Supplemental S4). There was mild heterogeneity for LTAs (*I* ^2^ = 16.6%) in 5 studies and severe heterogeneity for inotropic use in 3 studies (*I* ^2^ = 68.3%), respectively.

However, Intensive Care Unit (ICU) LOS was longer in the prolonged QTc group versus the normal QTc group (SMD 0.94, 95% CI 0.17–1.71, *p* = 0.0169) with severe heterogeneity between the two studies (*I* ^2^ = 85%), demonstrated in Supplemental S4. Moreover, positive pressure ventilation (PPV) was more frequently required in patients with prolonged QTc than in those with normal QTc (OR 2.41, 95% CI 1.06–5.44, *p* = 0.0349) with mild heterogeneity (*I* ^2^ = 21%) between the studies (Supplemental S4).

Sensitivity analysis was performed to address the moderate‐to‐severe heterogeneity identified for the all‐cause mortality, cardiogenic shock, and inotropic use outcomes. When excluding Song et al.^21^ from the cumulative all‐cause mortality *I* ^2^, the heterogeneity became mild (*I* ^2^ = 0%). This was likely secondary to longer mean follow‐up durations (4.2 and 4.8 years vs. 5.7 years). Moreover, when evaluating cardiogenic shock, removing Hohneck et al.^13^ reduced *I* ^2^ to 48.1% (mild heterogeneity). This was likely secondary to the distribution of the study population, which was significantly skewed toward prolonged QTc (73 prolonged QTc patients vs. 32 normal QTc patients), and composed of 33% of the composite prolonged QTc population evaluated within the meta‐analysis. Lastly, with respect to inotropic use, when excluding Song et al.,^21^ *I* ^2^ was reduced to 0% (mild heterogeneity). This was likely secondary to higher rates of cardiogenic shock compared to the other studies.

## 4 DISCUSSION

To our knowledge, this study is the largest, most comprehensive, and only meta‐analysis to evaluate clinical outcomes across three high‐risk arrhythmic subgroups in Takotsubo cardiomyopathy. We showed that the presence of atrial and life‐threatening arrhythmias resulted in significantly increased risk of in‐hospital mortality and cardiogenic shock in Takotsubo cardiomyopathy, which was not seen with prolonged QTc.

### 4.1 Atrial arrhythmias

Several studies have investigated the prevalence of atrial fibrillation in patients with Takotsubo cardiomyopathy ranging from 6.4% to 25%,^6^, ^23^, ^25^, ^27^, ^28^ and it remains the most common arrhythmia in TCM.^25^ Previous studies have also highlighted the detrimental effects of atrial arrhythmias on cardiovascular outcomes in TCM.^6^, ^25^, ^29^, ^30^, ^31^, ^32^ For instance, Stiermaier et al.^31^ reported that patients with TCM who developed atrial fibrillation experienced worse in‐hospital outcomes, including higher rates of cardiogenic shock and mortality. Furthermore, a previous meta‐analysis by Angeli et al.^33^ demonstrated that the risk of mortality doubles in atrial fibrillation patients.

The pathogenesis of atrial arrhythmias in TCM remains a significant area for further research. Although the exact mechanisms remain unclear, it is thought to be the interplay of inflammation, channelopathy, left ventricular dysfunction and stress, structural and functional changes in the atria that contribute toward the development of AAs.^9^, ^34^, ^35^, ^36^, ^37^, ^38^, ^39^ More specifically, the resultant detrimental hemodynamic changes from the loss of atrial contraction, disassociation of atrioventricular synchrony, and decreased diastolic filling time all provide the substrate for poor outcomes and complications.

There is still debate regarding the optimal management of AAs in TCM. Ahsan et al. identified a mortality benefit of beta‐blockade in TCM patients from the suppression of the catecholamine drive.^40^ However, these patients tend to have lower LVEFs (also found in our study), in which the use of beta‐blockade can further LV impairment. As there are no guidelines for the short‐term or long‐term management of AAs in TCM, the treatment is tailored to each individual's risk profile and clinical context.

### 4.2 Life‐threatening arrhythmias and QTc changes

Ventricular arrhythmias occur in 4–10% of cases^29^, ^41^ and are the most common form of LTA in TCM. They have been shown to contribute to significant morbidity, mortality, influence long‐term prognosis, and occur within the first 24 hours of diagnosis.^42^ Additionally, atrioventricular heart blocks and asystole are other less common causes of LTAs in patients with TCM.^20^, ^43^, ^44^ Previous studies have reported higher in‐hospital mortality and cardiogenic shock in patients with LTAs compared to those without.^20^, ^45^ Rathore et al. and Isogai et al. included patients with a Takotsubo diagnosis and LTAs on national‐level database studies relying on ICD‐10 codes, which are great for highlighting trends, but do not provide much clinical context.^41^, ^46^

The electrocardiographic changes have been well described in TCM. They start as transient q waves, ST‐segment elevations, and evolve into large T‐wave inversions and QTc interval prolongation.^47^, ^48^ Prolonged QTc is prevalent in TCM, affecting at least half of the patients.^12^ It is a known association with torsades de pointes, which can often self‐terminate, but can also degenerate into more malignant LTAs such as ventricular fibrillation.^49^ Interestingly, QTc prolongation in TCM can be apparent until 2 months after the index event.^48^, ^50^, ^51^

Dynamic changes in QTc have also been described in TCM.^45^ Madias et al.^22^ displayed a lengthening of the QTc until Day 2 after diagnosis of TCM in patients that developed LTAs, which eventually decreased to a similar value of patients that did not develop LTAs. Furthermore, there was a higher baseline QTc in the LTA group compared to the non‐LTA group. However, our study, like Jesel et al.,^20^ did not show a difference in baseline QTc between LTAs and non‐LTAs.

Prolonged QTc can occur in TCM due to focal myocarditis, oxidative stress, coronary vasospasm, and massive catecholamine release, which can all affect repolarization.^52^, ^53^, ^54^ Furthermore, other predisposing factors such as bradycardia, electrolyte abnormalities, and even congenital genetic mutations^55^ can increase the risk of QTc prolongation in patients with TCM.

Previous studies have been conflicting about the prognostic value of QTc prolongation in TCM. Madias and Imran et al. found that long QTc has a higher risk of ventricular arrhythmia, cardiogenic shock, and use of vasoactive agents.^12^, ^22^ Contrarily, Pinho et al.^18^ discovered that there was no statistically significant difference in long‐term and in‐hospital complications regardless of QTc, and Hohneck et al.^13^ even discovered a protective effect of long QTc for similar outcomes.

While our study did not find any statistically significant difference in mortality or cardiogenic shock, there was an increase in in‐hospital complications such as the ICU LOS and use of PPV. Although there was no statistical increase in the development of LTAs with prolonged QTc, it is still clinically relevant and demonstrates that there are both QTc‐dependent and QTc‐independent mechanisms that increase the risk of LTAs. Moreover, as QTc is dynamic in TCM, there could also be different time‐dependent triggers in the acute and chronic phases. Lastly, prolonged QTc can also be associated with other LTAs such as monomorphic ventricular tachycardia^56^, ^57^ and polymorphic ventricular tachycardia that is not torsades (pseudo‐torsades de pointes).^58^

Management of LTAs is tailored toward the specific presenting arrhythmia and associated hemodynamics. Beta‐blockade and Group III anti‐arrhythmics should be used cautiously in patients with QTc prolongation due to the risk of further lengthening, and short‐acting agents (esmolol) or temporary pacing (in concomitant bradycardia) can be considered instead.^59^ Although rare, these occurrences (such as complete heart block) may persist long‐term and occasionally require pacemaker placement.^60^ Guidance for defibrillator placement for secondary prevention is controversial and is largely from expert opinion, as the risk of recurrence of ventricular arrhythmias is unknown.^22^, ^42^, ^51^

### 4.3 Clinical implications

We conducted univariate analysis of effect modifiers to identify further risk factors that can confer worse clinical outcomes (Supplemental S6). In the case of atrial arrhythmias, increasing age lengthened in‐hospital stay by 0.1328 with a *p*‐value <0.0001. Interestingly, in the evaluation of the prolonged QTc, older age decreased all‐cause mortality (coefficient − 0.6118, *p* = 0.0314) and inotrope use (coefficient − 0.5354, *p* = 0.012), while ST‐Elevation increased all‐cause mortality (coefficient 0.041, *p* = 0.021). Furthermore, a history of hypertension decreased inotrope use (coefficient − 0.0494, *p* = 0.0162). Unfortunately, the paucity of data renders these conclusions unreliable, and univariate analysis could not be conducted on all the outcomes.

All uncomplicated TCM patients with prolonged QTc >490 ms should be transferred to the cardiac ICU due to the increased risk of ventricular arrhythmias for at least 48–72 h of monitoring.^61^, ^62^ De‐escalation of care should not occur until the resolution of QTc prolongation occurs.^63^ Our analysis suggests that a younger age and the presence of ST‐Elevation on EKG are negative prognostic factors in TCM patients with prolonged QTc. Although more studies are needed to investigate these effects, patients with these risk factors may warrant lower QTc thresholds (QTc >460 ms) for early escalation of care.

In conclusion, our findings shed new light on the intricate interplay between arrhythmias and outcomes in TCM. Our study lays the groundwork for developing more targeted risk assessment and treatment strategies by delineating the diverse impact of various arrhythmia subtypes.

### 4.4 Limitations and future directions

While this study provides valuable insights, it is essential to acknowledge its inherent limitations. Importantly, the definitions of prolonged QTc varied across studies. Due to the lack of clarity in clinical reporting, it is difficult to determine that the presence of QTc prolongation itself is predictive of torsades de pointes. Although Song et al.^21^ identify torsades de pointes as an independent outcome (of which there were none), others only report life‐threatening arrhythmias in totality. This implicitly creates inconsistency, as a patient could have had torsades de pointes that degenerated into ventricular fibrillation, which could have been only reported as a life‐threatening arrhythmia. Furthermore, despite having baseline QTc in the LTAs subgroup analysis and the difference being statistically insignificant, it is also important to acknowledge that TCM patients are known to have dynamic changes in QTc which can also trigger life‐threatening arrhythmias. Moreover, although the sample size was adequate for identifying significant associations, it may not capture the full spectrum of arrhythmic complications and granular clinical data (such as ST‐Elevation level related to QTc and/or average QTc between normal and prolonged QTc groups) in Takotsubo cardiomyopathy.

The retrospective data collection may introduce selection bias, hindering the establishment of causal relationships. Additionally, using the same patients in different stratifications across studies could overestimate the number of patients. Furthermore, drawing the study population from a single center brings the potential for selection bias, given the inclusion of single‐center studies and the absence of randomization, allowing for residual confounding factors. Additionally, the reliance on available medical records and EKG data for diagnosing arrhythmias and QTc prolongation may be subject to misclassification or incomplete documentation. Uncontrolled confounding factors, such as variations in treatment protocols and patient comorbidities, could impact the observed outcomes.

Although we used random effects models and conducted sensitivity analysis, it is crucial to acknowledge that high levels of heterogeneity (*I* ^2^ > 50%) of certain clinical outcomes make it difficult to ascertain the predictive value of pooled results, and these findings should be interpreted with caution. Given this study's observational nature, it is vital to emphasize the critical need for comprehensive, randomized, prospective studies with extended follow‐up periods to strengthen the evidence base.

Future multicenter prospective studies with standardized QTc monitoring protocols are necessary to evaluate treatment effects on clinical outcomes. Moreover, ongoing research to better understand the underlying QTc‐dependent and independent proarrhythmic mechanisms for LTAs and AAs is necessary to identify tailored antiarrhythmic therapies and secondary prevention strategies in this high‐risk patient population.

## 5 CONCLUSION

In this meta‐analysis, we have discovered that atrial arrhythmias and life‐threatening arrhythmias substantially impact the outcomes of individuals with Takotsubo cardiomyopathy, with significantly higher rates of cardiogenic shock, in‐hospital mortality, and prolonged lengths of stay. Furthermore, we found that QTc prolongation is linked to increased use of positive pressure ventilation and longer stays in the ICU. These findings emphasize the importance of tailoring monitoring and management strategies to address the specific arrhythmic profiles in Takotsubo patients and improve clinical outcomes.

## AUTHOR CONTRIBUTIONS

I certify that all authors listed above meet the authorship criteria and that all authors participated and are in agreement with the manuscript.

## CONFLICT OF INTEREST STATEMENT

The authors declare no conflict of interests for this article.

## Supporting information


Data S1:
