# Supplementary material for: Prognostic relevance of arrhythmic and QTc burden in takotsubo cardiomyopathy: A systematic review and meta‐analysis
Source: J Arrhythm. 2025 Jul 16;41(4):e70138. doi: 10.1002/joa3.70138 (PMC12267671; doi:10.1002/joa3.70138)
Supplement: Supplementary file 1 — Data S1: [file JOA3-41-e70138-s001.docx]

**Supplemental Files Index**

1. Supplemental S1: Preferred Reporting Items for Systematic Reviews and Meta-analyses (PRISMA) checklist
2. Supplemental S2: AMSTAR-2 (Assessing the methodological quality of systematic reviews-2) Guidelines checklist
3. Supplemental S3: Research Question, PICO, MeSH, Keywords and Search Strategy
4. Supplemental S4: Other Secondary outcomes of the study
5. Supplemental S5: Newcastle-Ottawa Scale for assessment of bias for observational studies
6. Supplemental S6: Meta-Regression of Effect Modifiers in QTc Changes, LTAs, and AAs
7. Supplemental S7: Definitions of prolonged QTc

**Supplemental S1: Preferred Reporting Items for Systematic Reviews and Meta-analyses (PRISMA) checklist**


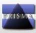
**PRISMA 2020 Checklist**

| **Section and**  **Topic** | **Item**  **#** | **Checklist item** | **Location**  **where item  is reported** |
| --- | --- | --- | --- |
| **TITLE** | | |  |
| Title | 1 | Identify the report as a systematic review. | X |
| **ABSTRACT** | | |  |
| Abstract | 2 | See the PRISMA 2020 for Abstracts checklist. | X |
| **INTRODUCTION** | | |  |
| Rationale | 3 | Describe the rationale for the review in the context of existing knowledge. | X |
| Objectives | 4 | Provide an explicit statement of the objective(s) or question(s) the review addresses. | X |
| **METHODS** | | |  |
| Eligibility criteria | 5 | Specify the inclusion and exclusion criteria for the review and how studies were grouped for the syntheses. | X |
| Information  sources | 6 | Specify all databases, registers, websites, organizations, reference lists and other sources searched or consulted to identify studies. Specify the date when each source was last searched or consulted. | X |
| Search strategy | 7 | Present the full search strategies for all databases, registers and websites, including any filters and limits used. | X |
| Selection process | 8 | Specify the methods used to decide whether a study met the inclusion criteria of the review, including how many reviewers screened each record and each report retrieved, whether they worked independently, and if applicable, details of automation tools used in the process. | X |
| Data collection  process | 9 | Specify the methods used to collect data from reports, including how many reviewers collected data from each report, whether they worked independently, any processes for obtaining or confirming data from study investigators, and if applicable, details of automation tools used in the process. | X |
| Data items | 10a | List and define all outcomes for which data were sought. Specify whether all results that were compatible with each outcome domain in each study were sought (e.g. for all measures, time points, analyses), and if not, the methods used to decide which results to collect. | X |
|  | 10b | List and define all other variables for which data were sought (e.g. participant and intervention characteristics, funding sources). Describe any assumptions made about any missing or unclear information. | X |
| Study risk of bias assessment | 11 | Specify the methods used to assess risk of bias in the included studies, including details of the tool(s) used, how many reviewers assessed each study and whether they worked independently, and if applicable, details of automation tools used in the process. | X |
| Effect measures | 12 | Specify for each outcome the effect measure(s) (e.g. risk ratio, mean difference) used in the synthesis or presentation of results. | X |
| Synthesis  methods | 13a | Describe the processes used to decide which studies were eligible for each synthesis (e.g. tabulating the study intervention characteristics and comparing against the planned groups for each synthesis (item #5)). | X |
|  | 13b | Describe any methods required to prepare the data for presentation or synthesis, such as handling of missing summary statistics, or data conversions. | x |
|  | 13c | Describe any methods used to tabulate or visually display results of individual studies and syntheses. | X |
|  | 13d | Describe any methods used to synthesize results and provide a rationale for the choice(s). If meta-analysis was performed, describe the model(s), method(s) to identify the presence and extent of statistical heterogeneity, and software package(s) used. | X |
|  | 13e | Describe any methods used to explore possible causes of heterogeneity among study results (e.g. subgroup analysis, meta-regression). | X |
|  | 13f | Describe any sensitivity analyses conducted to assess robustness of the synthesized results. | X |
| Reporting bias  assessment | 14 | Describe any methods used to assess risk of bias due to missing results in a synthesis (arising from reporting biases). |  |
| Certainty  assessment | 15 | Describe any methods used to assess certainty (or confidence) in the body of evidence for an outcome. | X |


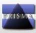
**PRISMA 2020 Checklist**

| **Section and**  **Topic** | **Item**  **#** | **Checklist item** | **Location**  **where item  is reported** |
| --- | --- | --- | --- |
| **RESULTS** | | |  |
| Study selection | 16a | Describe the results of the search and selection process, from the number of records identified in the search to the number of studies included in the review, ideally using a flow diagram. | X |
|  | 16b | Cite studies that might appear to meet the inclusion criteria, but which were excluded, and explain why they were excluded. | X |
| Study  characteristics | 17 | Cite each included study and present its characteristics. | X |
| Risk of bias in  studies | 18 | Present assessments of risk of bias for each included study. | X |
| Results of  individual studies | 19 | For all outcomes, present, for each study: (a) summary statistics for each group (where appropriate) and (b) an effect estimates and its precision (e.g. confidence/credible interval), ideally using structured tables or plots. | X |
| Results of  syntheses | 20a | For each synthesis, briefly summarize the characteristics and risk of bias among contributing studies. | X |
|  | 20b | Present results of all statistical syntheses conducted. If meta-analysis was done, present for each the summary estimate and its precision (e.g.  confidence/credible interval) and measures of statistical heterogeneity. If comparing groups, describe the direction of the effect. | X |
|  | 20c | Present results of all investigations of possible causes of heterogeneity among study results. | X |
|  | 20d | Present results of all sensitivity analyses conducted to assess the robustness of the synthesized results. |  |
| Reporting biases | 21 | Present assessments of risk of bias due to missing results (arising from reporting biases) for each synthesis assessed. | X |
| Certainty of  evidence | 22 | Present assessments of certainty (or confidence) in the body of evidence for each outcome assessed. | X |
| **DISCUSSION** | | |  |
| Discussion | 23a | Provide a general interpretation of the results in the context of other evidence. | X |
|  | 23b | Discuss any limitations of the evidence included in the review. | X |
|  | 23c | Discuss any limitations of the review processes used. | X |
|  | 23d | Discuss implications of the results for practice, policy, and future research. | X |
| **OTHER INFORMATION** | | |  |
| Registration and protocol | 24a | Provide registration information for the review, including register name and registration number, or state that the review was not registered. |  |
|  | 24b | Indicate where the review protocol can be accessed, or state that a protocol was not prepared. |  |
|  | 24c | Describe and explain any amendments to information provided at registration or in the protocol. |  |
| Support | 25 | Describe sources of financial or non-financial support for the review, and the role of the funders or sponsors in the review. | X |
| Competing  interests | 26 | Declare any competing interests of review authors. | X |
| Availability of  data, code and  other materials | 27 | Report which of the following are publicly available and where they can be found: template data collection forms; data extracted from included studies; data used for all analyses; analytic code; any other materials used in the review. | X |

*From:* Page MJ, McKenzie JE, Bossuyt PM, Boutron I, Hoffmann TC, Mulrow CD, et al. The PRISMA 2020 statement: an updated guideline for reporting systematic reviews. BMJ 2021;372:n71. doi:  10.1136/bmj.n71

For more information, visit: <http://www.prisma-statement.org/>

**Supplemental S2: AMSTAR-2 (Assessing the methodological quality of systematic reviews-2) Guidelines checklist**

**
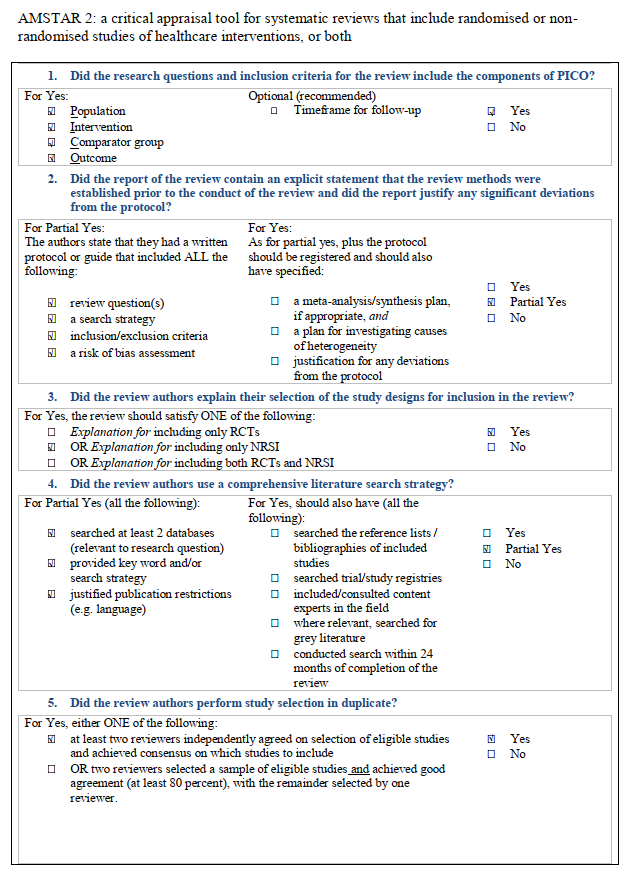
**

**
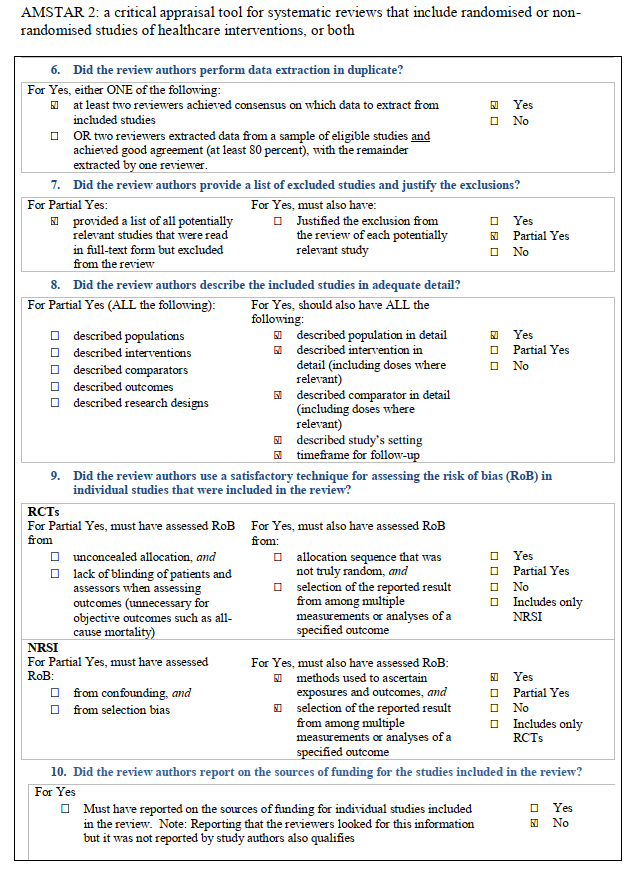
**
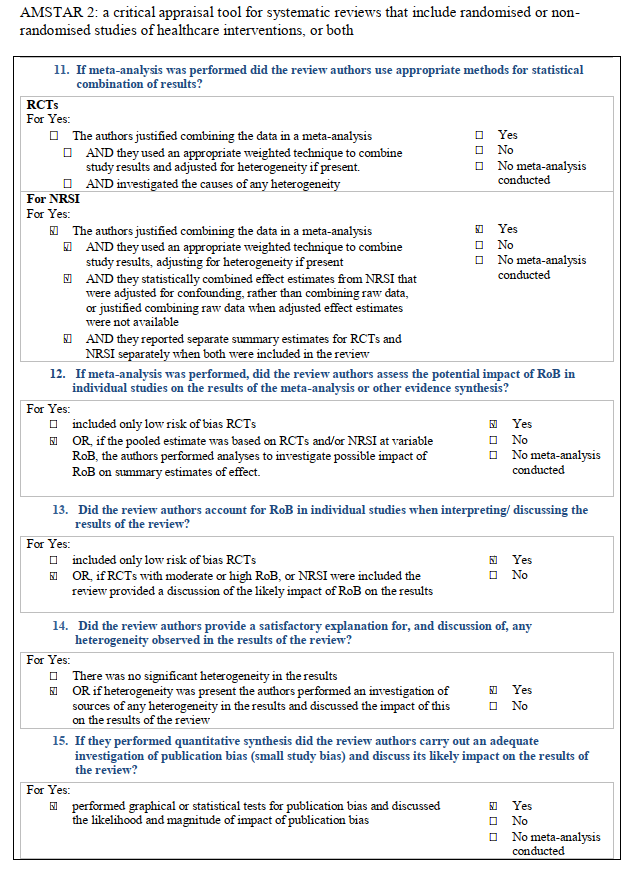

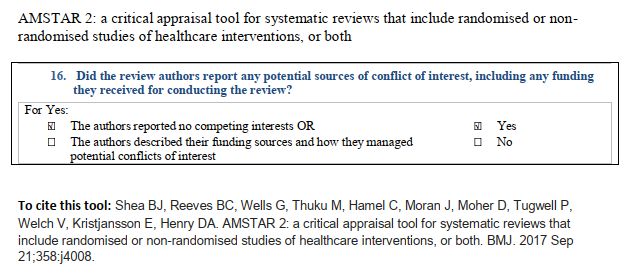


**Supplemental S3: Research Question, PICO, MeSH, Keywords, and Search Strategy**

**Research Question:** *Prognostic Relevance of Arrhythmic and QTc Burden in Takotsubo Cardiomyopathy*

**PICO:**

**Population:** Patients with Takotsubo cardiomyopathy

**Intervention:** Presence of arrhythmia or long QTc

**Comparison:** No arrhythmia or normal QTc

**Outcome:**

**1)** **Primary outcomes** *included* *in-hospital* *mortality*

**2) Secondary outcomes** *included use of inotropes, intra-arterial balloon pump (IABP) use, length of stay (LOS), positive pressure ventilation (PPV), thromboembolism***,** *cardiogenic shock, life-threatening arrhythmias, long-term mortality*

**Study type:** Odds ratio to compare binary outcomes and standard mean difference to compare continuous outcomes meta-analyses.

**MeSH Terms & Keywords:**

Takotsubo Cardiomyopathy

Broken Heart Syndrome

Apical Ballooning Syndrome

Stress Cardiomyopathy

Arrhythmias

Torsades de Pointes

Prolonged QTc

Prognosis

Mortality

Outcomes

**Detailed search strategy for each of the included databases.**

| **Database** | **Search Strategy** | **Articles retrieved** |
| --- | --- | --- |
| PubMed | ("Takotsubo Cardiomyopathy"[Mesh] OR "Takotsubo Syndrome"[Mesh] OR "Broken Heart Syndrome" OR "Takotsubo" OR "Apical Ballooning Syndrome" OR "Stress Cardiomyopathy") AND ("Arrhythmias, Cardiac"[Mesh] OR "Cardiac Arrhythmias" OR "Heart Arrhythmia" OR "Arrhythmia" OR "Atrial Fibrillation" OR "Ventricular Tachycardia" OR “Torsades de Pointes” OR "Ventricular Fibrillation" OR "Bradycardia" OR "Tachycardia") OR ("QTc Interval"[Mesh] OR "QT Interval" OR "Prolonged QT Interval" OR "QTc Prolongation")  AND ("Prognosis"[Mesh] OR "Prognostic" OR "Outcome" OR "Mortality" OR "Survival" OR "Predictive Value" OR "Prognostic Value") | 481 |
| Embase | ('takotsubo cardiomyopathy'/exp OR 'takotsubo syndrome'/exp OR 'broken heart syndrome' OR 'takotsubo' OR 'apical ballooning syndrome' OR 'stress cardiomyopathy') AND ('arrhythmia'/exp OR 'cardiac arrhythmia' OR 'heart arrhythmia' OR 'atrial fibrillation' OR 'ventricular tachycardia' OR ‘torsades de pointes’ OR 'ventricular fibrillation' OR 'bradycardia' OR 'tachycardia' OR  'qt interval'/exp OR 'qt interval' OR 'prolonged qt interval' OR 'qtc prolongation') AND ('prognosis'/exp OR 'prognostic' OR 'outcome' OR 'mortality' OR 'survival' OR 'predictive value' OR 'prognostic value') | 401 |

**Supplemental S4: Secondary outcomes**

**
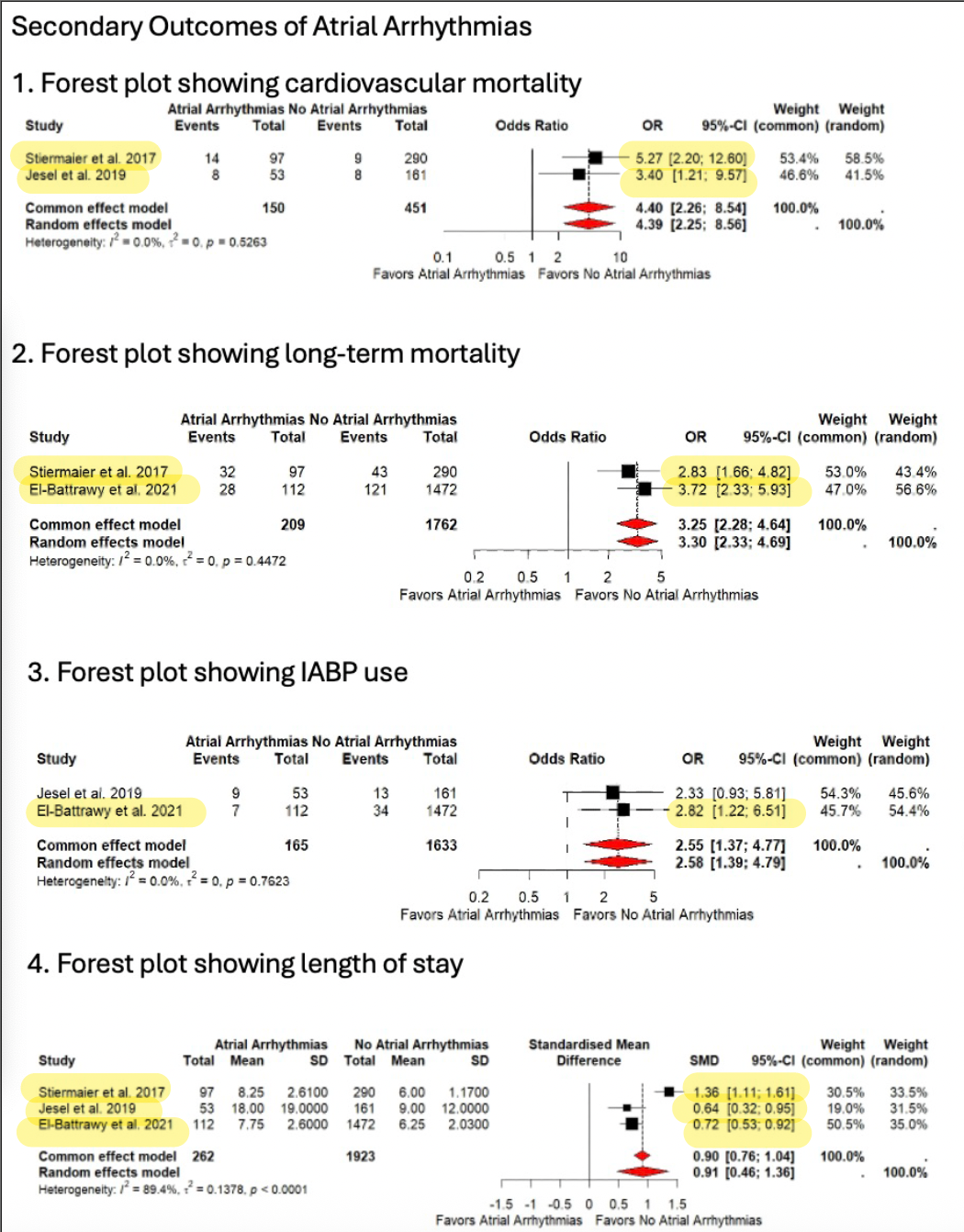
**

**
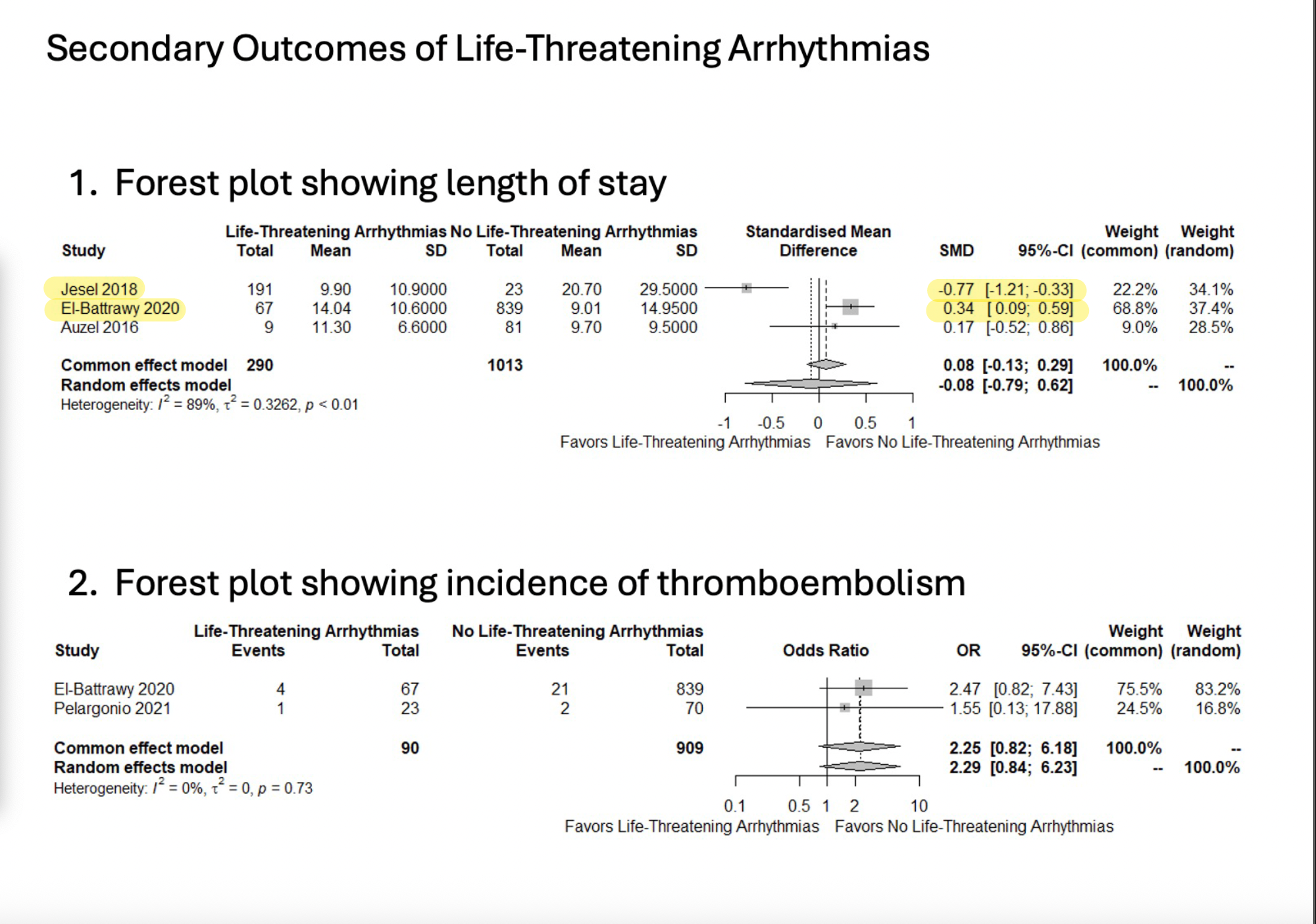
**

**
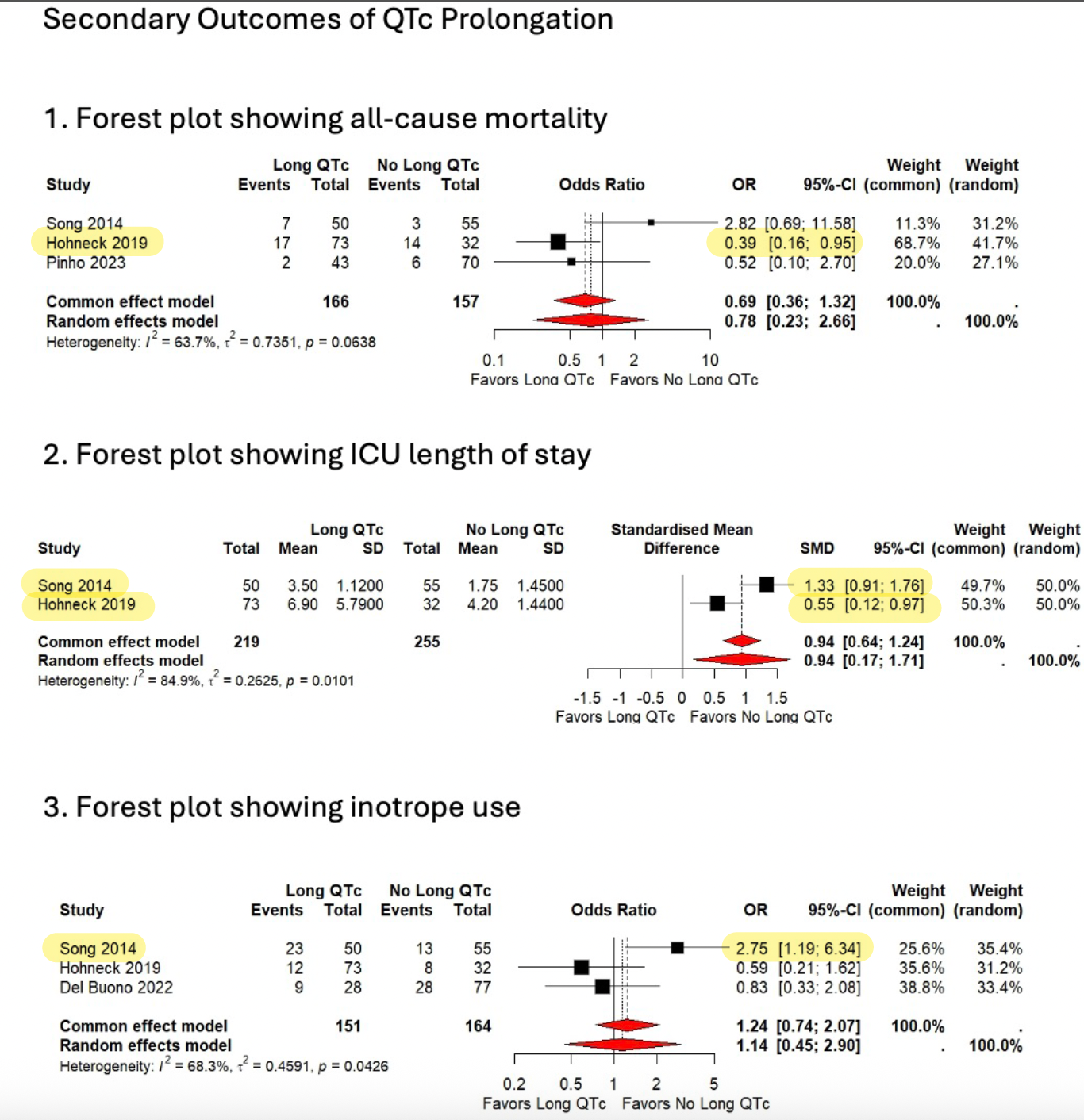
**

**
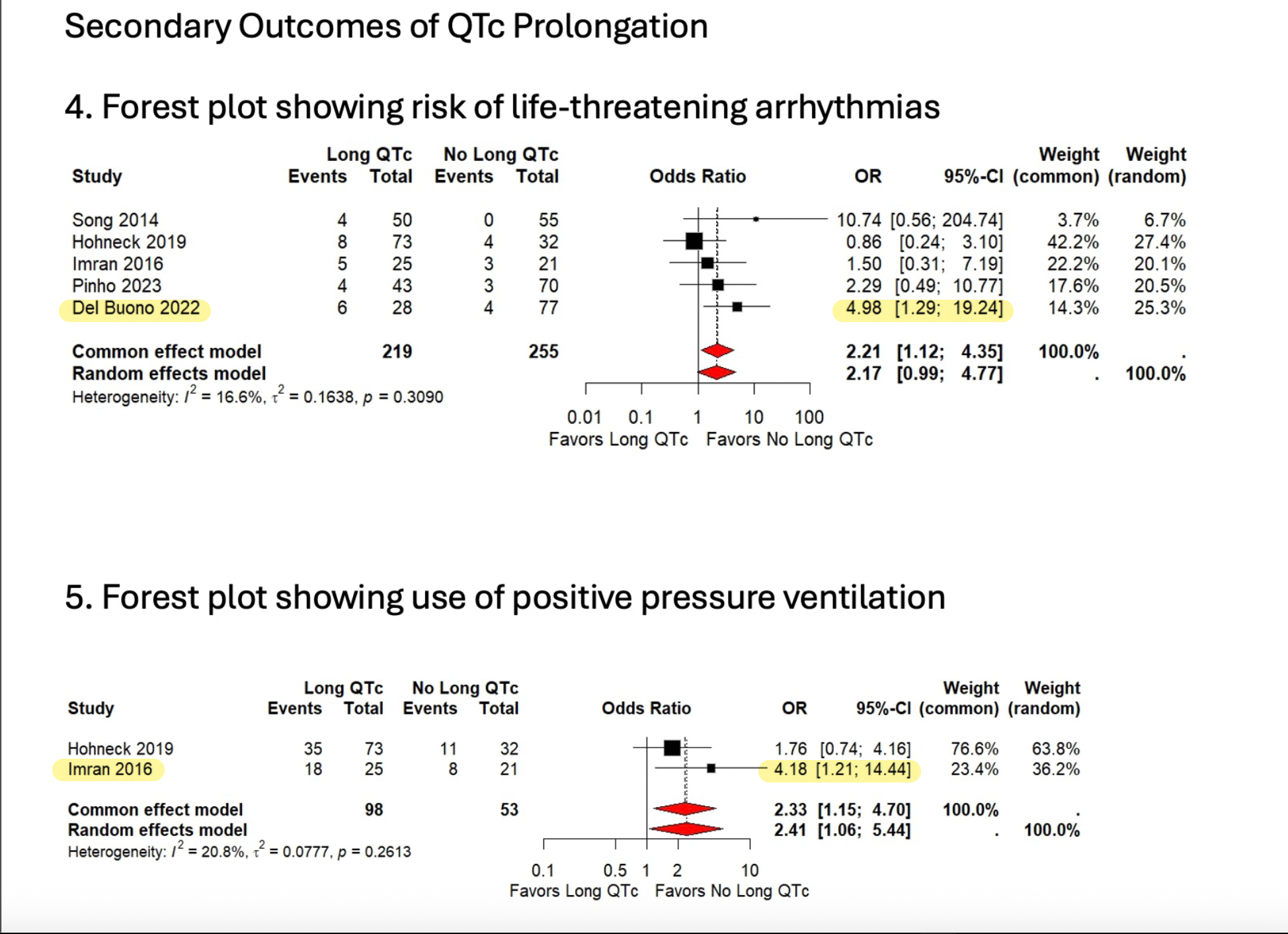
**

**Supplemental S5: Newcastle-Ottawa Scale for assessment of bias for observational studies**

| Study | Representativeness of the exposed cohort | Selection of the non-exposed cohort | Ascertainment of exposure | Demonstration that outcome of interest was not present at start of study | Comparability of cohorts on the basis of the design or analysis | Assessment of outcome | Was follow-up long enough for outcomes to occur | Adequacy of follow-up cohort | Total |
| --- | --- | --- | --- | --- | --- | --- | --- | --- | --- |
| Auzel 2016 | 1 | 1 | 1 | 1 | 2 | 1 | 1 | 0* | 8/9 |
| El-Battrawy 2020. | 1 | 1 | 1 | 1 | 2 | 1 | 1 | 1 | 9/9 |
| El-Battrawy 2021 | 1 | 1 | 1 | 1 | 2 | 1 | 1 | 1 | 9/9 |
| Hohneck 2018 | 1 | 1 | 1 | 1 | 2 | 1 | 1 | 1 | 9/9 |
| Imran 2016 | 1 | 1 | 1 | 1 | 2 | 1 | 1 | 0* | 8/9 |
| Jesel 2018 | 1 | 1 | 1 | 1 | 2 | 1 | 1 | 1 | 9/9 |
| Jesel 2019 | 1 | 1 | 1 | 1 | 2 | 1 | 1 | 1 | 9/9 |
| Madias 2011 | 1 | 1 | 1 | 1 | 2 | 1 | 1 | 1 | 9/9 |
| Pelargonio 2021 | 1 | 1 | 1 | 1 | 2 | 1 | 1 | 1 | 9/9 |
| Pinho 2023 | 1 | 1 | 1 | 1 | 2 | 1 | 1 | 1 | 9/9 |
| Song 2013 | 1 | 1 | 1 | 1 | 2 | 1 | 1 | 1 | 9/9 |
| Stiermaier 2017 | 1 | 1 | 1 | 1 | 1 | 1 | 1 | 0* | 7/9 |
| Del Buono 2022 | 1 | 1 | 1 | 1 | 1 | 1 | 1 | 0* | 7/9 |

* = no follow-up mentioned

**Supplemental S6: Meta-Regression of Effect Modifiers in QTc Changes, LTAs, and AAs**


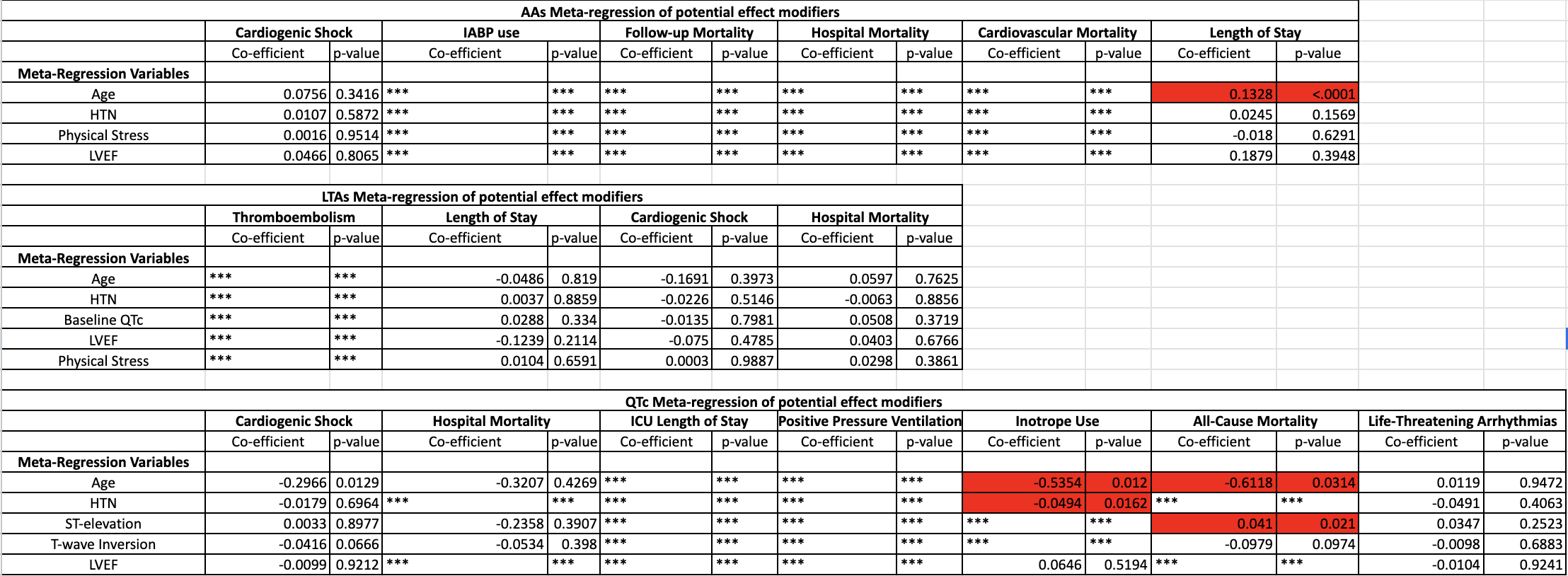


**Supplemental S7: Definitions for Prolonged QTc**

| Imran 2016 | QTc > 470 ms (males) and QTc > 480 (females) |
| --- | --- |
| Pinho 2023 | QTc > 460 ms |
| Del Buono 2022 | QTc > 460 ms |
| Hohneck 2018 | QTc > 450 ms (males) and QTc > 460 ms (females) |
| Song 2014 | QTc > 430 ms (males) and QTc > 450 ms (females) |

QTc = Corrected QT interval, ms = milliseconds
